# Editorial: Cholesterol and cancer drug resistance: Molecular, signaling, and therapeutic aspects

**DOI:** 10.3389/fgene.2022.994181

**Published:** 2022-09-15

**Authors:** Mandeep Kaur, Zodwa Dlamini

**Affiliations:** ^1^ School of Molecular and Cell Biology, University of the Witwatersrand, Johannesburg, South Africa; ^2^ Pan African Cancer Research Institute (PACRI), University of Pretoria, Pretoria, South Africa

**Keywords:** cholesterol, cancer drug resistance, molecular pathways, EMT, therapeutic targets

Cancer was responsible for approximately 10 million deaths in 2020. Despite the notable progress made in anticancer therapy, cancer patients present with *de novo* or acquired resistance, which plays a key role in relapse. Several well-defined biological processes, including but not limited to impaired DNA damage repair, increased efflux of drugs, and epithelial-mesenchymal transition (EMT), have been linked to drug resistance in cancer cells. Over the past decade, accumulating evidence has focused on the accumulation of cholesterol in cancer cells as one of the driving mechanisms of drug resistance. This Research Topic explored molecular pathways responsible for cancer drug resistance in a range of cancers, especially narrowing down to pathways impacted by cholesterol while suggesting the role of cholesterol-lowering agents as potential anticancer therapeutics.

Several growth factor signaling pathways [involving insulin-like growth factor (IGF), vascular endothelial growth factor (VEGF), epidermal growth factor (EGF), fibroblast growth factor (FGF), transforming growth factor-beta (TGF-β), connective tissue growth factor, nerve growth factor (NGF), and hepatocyte growth factor (HGF)] are responsible for pancreatic cancer (PC) initiation and progression. These pathways are critical in various biological processes such as cell differentiation, migration, proliferation, and apoptosis. Their cellular functions provide unique opportunities to target them toward pancreatic ductal adenocarcinoma (PDAC) treatment. In recent years, several attempts have been made to do so, however, with some challenges. Xelwa et al. discussed some of the opportunities and challenges that emanate from targeting growth factors. Some of these challenges arise from the heterogeneity of the tumor and its ability to evade detection and destruction. Subsequently, the authors have highlighted the potential of the combinatorial targeting of the growth factor signaling pathways as an avenue to bypass these challenges, especially to combat drug resistance in PC.

The well-known signaling protein Akt, a serine/threonine kinase has been hailed as a cell survival protein, in its phosphorylated form (phospho-Akt) activates several downstream proteins involved in the process such as cell proliferation, migration, invasion, metabolism, and apoptosis. Thus, targeting dysregulated Akt in cancer cells could be effective in disease prevention and therapy. Kumar and Mandal argued that cholesterol-lowering drugs such as statins (lovastatin) in combination with selective cholesterol absorption inhibitors (ezetimibe) and citrate lyase inhibitors (bempedoic acid, fibrates (fenofibrate) have shown anticancer activity in various cancers (breast, colorectal, liver, pancreatic, prostate, and others) by modifying operative signaling pathways such as PTEN/Akt, PI3k/Akt, Akt/NF-κB, Akt/FOXO1, and Akt/mTOR. The authors have substantiated the significance of cholesterol in cancer cells by tabulating the past and present clinical trials involving statins as anticancer therapeutics. The authors have argued in the light of the published literature (see references in the article) that inhibition of Akt using cholesterol-lowering drugs could improve the chemosensitivity of cancer cells, thus mitigating therapy resistance and metastasis.

Emerging scientific evidence implicates cholesterol dyshomeostasis in cancer progression and drug resistance. Importantly, the tumor cell plasticity program “EMT” plays a crucial role in drug resistance. Abdulla et al. attempted to delineate the complexities governing these two processes and partly addressed the gap in the literature with a specific focus on the influence of hypoxia on cellular cholesterol content and the resulting implications this has on EMT and drug resistance. The role of cholesterol in modulating multidrug-resistant transporters and transcriptional regulation of EMT has been explained in depth. The authors postulated a potential hypothesis that an intricate relationship exists between hypoxia, EMT, and cholesterol and consequently proposed that targeting cellular cholesterol could serve as a novel avenue to combat cancer pathogenesis.

While the abovementioned articles focused on using synthetic drugs such as statins as an anticancer therapy, it is important to mention that about 30% of the patient population is non-responsive to statin therapy in general. Therefore, alternative cholesterol-lowering agents need to be identified and developed. Laka et al. accumulated evidence that phytochemicals may be used for lowering cholesterol by especially targeting the P53 pathway in cancer cells, where mutant *p53* is responsible for regulating cholesterol biosynthesis mevalonate pathway by activating Sterol regulatory element-binding protein 2 (SREBP-2) in cancer cells and wild-type *p53* represses SREBP-2 transcriptional activity. Several phytochemicals and their roles in cholesterol-lowering and cancer have been detailed in this article.

The articles published in this special topic corroborated the role of cholesterol in cancer and documented the published literature to explore the complex molecular pathways underlying cancer proliferation, drug resistance, and metastasis. There is a need to identify new classes of cholesterol-lowering compounds (both synthetic and natural products), and their testing as single agents or in combination with existing anticancer therapies must be done to restore drug sensitivity. Future studies need to focus on delineating the role of cholesterol in influencing the tumor microenvironment. It would be intriguing to recognize the role of cholesterol in T-cell exhaustion induced by the tumor microenvironment to potentially improve immunotherapies.

